# National trends in hypertension stratified by central adiposity using waist-to-height ratio in South Korea, 2005 to 2022

**DOI:** 10.1097/MD.0000000000043773

**Published:** 2025-08-15

**Authors:** Jaehyun Kong, Hyeseung Lee, Hyunjee Kim, Yesol Yim, Sooji Lee, Seohyun Hong, Lee Smith, Damiano Pizzol, Jaewon Kim, Ho Geol Woo, Jiyoung Hwang, Dong Keon Yon

**Affiliations:** a Center for Digital Health, Medical Science Research Institute, Kyung Hee University Medical Center, Kyung Hee University College of Medicine, Seoul, South Korea; b Department of Medicine, Kyung Hee University College of Medicine, Seoul, South Korea; c Department of Precision Medicine, Kyung Hee University College of Medicine, Seoul, South Korea; d Centre for Health, Performance and Wellbeing, Anglia Ruskin University, Cambridge, UK; e Department of Public Health, Faculty of Medicine, Biruni University, Istanbul, Turkey; f Health Unit, ENI, Maputo Mozambique; g Health Unit, ENI, San Donato Milanese, Italy; h Department of Neurology, Kyung Hee University Medical Center, Kyung Hee University College of Medicine, Seoul, South Korea; i Department of Pediatrics, Kyung Hee University College of Medicine, Seoul, South Korea.

**Keywords:** epidemiology, hypertension, obesity, Republic of Korea, waist-height ratio

## Abstract

Despite the growing prevalence of hypertension due to lifestyle changes and aging populations, with central obesity as a critical risk factor, there is limited research examining the association between central adiposity and hypertension trends. To address this gap, this study analyzed trends in hypertension prevalence among Korean adults categorized by healthy and increased central adiposity based on waist-to-height ratio (WHtR) and investigated the associated risk factors. We utilized data from 98,396 adults aged 30 years and older from the Korea National Health and Nutrition Examination Survey from 2005 to 2022 in South Korea. Participants were categorized into 2 groups according to central adiposity: low-WHtR group (<0.50) and high-WHtR group (≥0.50). Long-term trends in hypertension and adjusted odds ratios were analyzed, with stratified analyses by sociodemographic factors. Weighted linear regression and binary logistic regression were used to calculate β coefficients, β_diff_, and adjusted odds ratios with 95% confidence intervals. From 2005 to 2019, the prevalence of hypertension increased in both low and high-WHtR groups, with a more pronounced increase in the increased central obesity group. Notably, the prevalence was higher in males than in females in the low-WHtR group (20.47% in males vs 12.33% in females), but similar in both sexes in the high-WHtR group (43.44% vs 41.74%). Furthermore, subgroup analyses showed that females aged 30–59 years in the high-WHtR group had higher odds of hypertension than males, whereas males aged 60 years and older had higher odds than females. This study suggests that the association between central obesity and hypertension risk varies significantly across age and sex categories, highlighting the necessity for age- and sex-specific prevention and management strategies.

## 1. Introduction

As populations age and lifestyle patterns shift, hypertension has emerged as an increasingly prevalent global health challenge.^[1]^ Worldwide, it is estimated that 1.28 billion adults aged 30 to 79 years are affected by hypertension, of which 46% are unaware of their condition.^[1]^ Hypertension has been consistently associated with increased morbidity across multiple organ systems, particularly affecting cardiovascular, kidney, and cerebrovascular health.^[2]^ In South Korea, the prevalence of hypertension has been steadily rising, driven by population aging and the adoption of Western dietary habits. It remains a significant contributor to mortality from cardiovascular disease.^[3]^ The impact of hypertension extends beyond individual health outcomes to create significant societal costs, highlighting the imperative for comprehensive prevention and management approaches.^[4]^

Body mass index (BMI) has limitations in predicting hypertension risk and waist-to-height ratio (WHtR) is recognized as a better predictor.^[5]^ Compared to BMI, WHtR assesses central obesity based on waist circumference in relation to height and is known to be a more accurate predictor of disease risk transcending sex and age differences.^[6,7]^ In particular, central obesity is recognized as an important factor in increasing the risk of cardiovascular disease and metabolic syndrome due to the accumulation of visceral fat.^[8,9]^ However, there is a lack of studies that have observed trends in hypertension or analyzed risk factors for hypertension by WHtR.

Thus, this study aimed to investigate temporal trends in the prevalence of hypertension based on data from a large-scale and population-based serial study of Korean adults from 2005 to 2022, stratified by low and high-WHtR groups. We also analyzed the association between central obesity and hypertension by socioeconomic and behavioral factors, including sex and age group. This will help identify high-risk groups by sex and age, providing evidence to inform strategies for the prevention and management of hypertension.

## 2. Methods

### 2.1. Survey design and participants

This study utilized data from the Korea National Health and Nutrition Examination Survey (KNHANES), conducted by the Korea Disease Control and Prevention Agency (KDCA) from 2005 to 2022. KNHANES employed a detailed sampling framework based on the most recent population and housing census data. Participants aged 30 years or older were included in the analysis.^[1]^ Data were collected for each participant on variables including sex, age, region of residence, central adiposity group, BMI group, educational level, household income, and smoking status.^[10]^ Participants who had missing values or did not meet the age criterion were excluded (Fig. S1, Supplemental Digital Content, https://links.lww.com/MD/P629). A total of 98,396 participants, representative of the national population, were selected to assess and stratify the prevalence of hypertension according to the central adiposity group. Participants were distributed across the following survey periods: 35,005 individuals from 2005 to 2009, 15,610 from 2010 to 2012, 13,440 from 2013 to 2015, 20,601 from 2016 to 2019, and 13,740 from 2020 to 2022.

The research protocol was approved by the Institutional Review Boards of the KDCA (2007-02CON-04-P, 2008-04EXP-01-C, 2009-01CON-03-2C, 2010-02CON-21-C, 2011-02CON-06-C, 2012-01EXP-01-2C, 2013-07CON-03-4C, 2013-12EXP-035C, 2018-01-03-P-A, 2018-01-03-C-A, 2018-01-03-2C-A, 2018-01-03-5C-A, 2018-01-03-4C-A, 2022-11-16-R-A). Written informed consent was obtained from all participants prior to their inclusion in the study. Furthermore, KNHANES data, which are publicly available, serve as a valuable resource for diverse epidemiological research. The study was conducted in full compliance with the principles outlined in the Declaration of Helsinki.

### 2.2. Definition of hypertension and WHtR groups

We investigated the trends in hypertension prevalence among individuals aged 30 years and older, stratified by central adiposity groups. Blood pressure measurements were conducted following established protocols, and consistent with previous methodological approaches, individuals under 30 years of age were excluded from the analysis to maintain analytical consistency and comparability.^[1,11]^ Participants remained seated in a resting position for a minimum of 5 minutes, after which researchers ensured the proper placement of the blood pressure cuff on their arm. Researchers measured blood pressure 2 to 3 times on the participant’s right arm, and the second measurement or the average of the second and third measurements was used for the analysis. According to the 2024 European Society of Cardiology Guideline, hypertension was defined as systolic blood pressure ≥ 140 mm Hg, diastolic blood pressure ≥ 90 mm Hg, or the use of antihypertensive medication.^[1,12,13]^

WHtR, obtained by dividing waist circumference by height, was utilized to classify participants into 2 categories: low-WHtR group (WHtR < 0.50) and high-WHtR group (WHtR ≥ 0.50).^[5,14,15]^

### 2.3. Covariates

In this study, the analysis included the following covariates: age (30–39, 40–49, 50–59, and ≥ 60 years), region of residence (urban or rural), BMI group (underweight or normal [<22.9 kg/m²], overweight [23.0–24.9 kg/m²], obese [≥25.0 kg/m²], and unknown), education level (middle school or lower, and high school or higher), household income (lowest and second quartile, and third and highest quartile), and smoking status (current or ex-, and nonsmoker). The classification of region of residence was based on survey responses.^[16]^ BMI groups were defined according to the Asia-Pacific guidelines, and household income was categorized into standardized income quartiles using the sample household and population data from KNHANES.^[17]^

### 2.4. Statistical analyses

We conducted a weighted complex sampling analysis to calculate the national prevalence of hypertension by central adiposity groups. Weighted analyses were applied to determine hypertension prevalence rates. Linear and binary logistic regression models were employed to estimate β coefficients and adjusted odds ratios (aORs), both presented with 95% confidence intervals (CIs).^[18]^ To evaluate the impact of the COVID-19 pandemic, trends from 2005 to 2019 were compared with those from 2020 to 2022 using β differences (β_diff_) and their corresponding 95% CIs. Stratified analyses were performed across subgroups defined by variables such as sex, age, region of residence, BMI, education level, household income, and smoking status. Adjustments were applied to all covariates, with the exception of those used as stratification variables. Additionally, adjusted odds ratios for hypertension prevalence were calculated according to central adiposity groups, with adjustments made for all other covariates not used as stratification variables. Statistical analyses were performed using SAS software (version 9.4, SAS Institute, Cary). A 2-sided test was employed, with a *P*-value of < 0.05 considered statistically significant.^[19,20]^

## 3. Results

Table 1 presents the weighted characteristics of the 98,396 participants (Fig. S1, Supplemental Digital Content, https://links.lww.com/MD/P629). The weighted proportions of males and females were 48.76% (95% CI: 48.44–49.08) and 51.24% (50.92–51.56), respectively. Regarding central adiposity status, 46.47% (45.93–47.00) of participants were classified as low-WHtR, whereas 53.53% (53.00–54.07) had high-WHtR.

**Table 1:** Weighted characteristics of Korean adults based on the data obtained from the KNHANES from 2005 to 2022 (n = 98,396).

Variables	Total	Pre-pandemic				During the pandemic
		2005–2009	2010–2012	2013–2015	2016–2019	2020–2022
Overall, n	98,396	35,005	15,610	13,440	20,601	13,740
Sex, weighted % (95% CI)						
Male	48.76 (48.44–49.08)	49.05 (48.39–49.71)	48.68 (47.96–49.40)	47.85 (47.07–48.63)	48.78 (48.16–49.41)	49.36 (48.58–50.13)
Female	51.24 (50.92–51.56)	50.95 (50.29–51.61)	51.32 (50.60–52.04)	52.15 (51.37–52.93)	51.22 (50.59–51.84)	50.64 (49.87–51.42)
Age, yr, weighted % (95% CI)						
30–39	23.52 (22.95–24.09)	28.28 (26.93–29.63)	25.87 (24.57–27.18)	23.62 (22.29–24.95)	21.69 (20.56–22.83)	20.01 (18.72–21.30)
40–49	25.43 (24.94–25.92)	28.31 (27.20–29.41)	26.95 (25.73–28.16)	25.51 (24.40–26.63)	24.48 (23.55–25.42)	23.02 (21.84–24.20)
50–59	23.20 (22.75–23.65)	20.27 (19.40–21.15)	22.56 (21.52–23.60)	24.10 (23.03–25.17)	24.21 (23.33–25.08)	23.95 (22.82–25.09)
≥60	27.86 (27.31–28.40)	23.14 (22.15–24.14)	24.62 (23.51–25.73)	26.77 (25.58–27.95)	29.61 (28.45–30.78)	33.02 (31.58–34.47)
Region of residence, weighted % (95% CI)						
Urban	81.58 (80.26–82.90)	79.13 (76.18–82.08)	78.34 (74.89–81.80)	81.49 (78.46–84.52)	83.83 (81.28–86.37)	83.51 (80.56–86.46)
Rural	18.42 (17.10–19.74)	20.87 (17.92–23.82)	21.66 (18.20–25.11)	18.51 (15.48–21.54)	16.17 (13.63–18.72)	16.49 (13.54–19.44)
Central obesity†, weighted % (95% CI)						
Low-WHtR group	46.47 (45.93–47.00)	50.89 (49.72–52.06)	48.59 (47.30–49.88)	49.08 (47.77–50.38)	44.65 (43.62–45.69)	41.26 (40.01–42.51)
High-WHtR group	53.53 (53.00–54.07)	49.11 (47.94–50.28)	51.41 (50.12–52.70)	50.92 (49.62–52.23)	55.35 (54.31–56.38)	58.74 (57.49–59.99)
BMI group‡, weighted % (95% CI)						
Underweight or normal weight	39.22 (38.79–39.65)	37.67 (36.76–38.58)	40.72 (39.69–41.75)	41.08 (40.07–42.09)	39.56 (38.72–40.39)	37.06 (36.04–38.08)
Overweight	23.74 (23.38–24.10)	23.32 (22.58–24.07)	24.28 (23.42–25.14)	24.47 (23.62–25.31)	23.81 (23.11–24.51)	22.87 (22.00–23.74)
Obese	35.38 (34.96–35.81)	31.72 (30.85–32.60)	34.55 (33.53–35.56)	34.37 (33.43–35.30)	36.29 (35.46–37.11)	38.67 (37.60–39.75)
Unknown	1.66 (1.58–1.75)	7.28 (6.90–7.67)	0.45 (0.30–0.61)	0.09 (0.02–0.15)	0.35 (0.25–0.46)	1.40 (1.17–1.63)
Level of education, weighted % (95% CI)						
Elementary school or lower education	17.26 (16.81–17.70)	23.04 (21.97–24.10)	20.95 (19.83–22.07)	18.11 (17.01–19.20)	14.90 (14.06–15.74)	11.84 (10.95–12.73)
Middle school	10.95 (10.65–11.25)	12.98 (12.30–13.66)	12.43 (11.71–13.16)	11.24 (10.55–11.93)	10.18 (9.60–10.76)	8.82 (8.17–9.47)
High school	31.78 (31.28–32.29)	34.17 (33.05–35.30)	33.09 (31.90–34.29)	32.18 (31.00–33.36)	30.14 (29.16–31.11)	30.56 (29.41–31.71)
College or higher education	40.01 (39.28–40.73)	29.81 (28.36–31.25)	33.53 (31.99–35.07)	38.48 (36.90–40.06)	44.78 (43.27–46.29)	48.78 (47.07–50.49)
Household income, weighted % (95% CI)						
Lowest quartile	16.37 (15.89–16.85)	17.58 (16.50–18.65)	17.50 (16.37–18.62)	16.55 (15.37–17.72)	16.12 (15.15–17.08)	14.64 (13.59–15.69)
Second quartile	24.69 (24.16–25.21)	24.80 (23.65–25.94)	27.51 (26.18–28.84)	24.45 (23.20–25.69)	24.28 (23.27–25.28)	22.86 (21.66–24.05)
Third quartile	28.84 (28.31–29.38)	28.42 (27.26–29.57)	28.11 (26.94–29.29)	29.20 (27.82–30.58)	28.75 (27.74–29.77)	29.63 (28.35–30.90)
Highest quartile	30.10 (29.36–30.84)	29.21 (27.52–30.90)	26.88 (25.44–28.32)	29.80 (28.03–31.57)	30.86 (29.39–32.32)	32.88 (31.07–34.69)
Smoking status, weighted % (95% CI)						
Smoker	21.70 (21.31–22.09)	24.16 (23.39–24.92)	25.71 (24.74–26.68)	22.30 (21.36–23.23)	20.33 (19.56–21.10)	17.53 (16.65–18.42)
Ex-smoker	22.98 (22.64–23.32)	20.11 (19.43–20.80)	21.31 (20.52–22.10)	21.32 (20.53–22.10)	23.98 (23.33–24.63)	26.83 (25.98–27.68)
Nonsmoker	54.34 (53.95–54.73)	49.70 (48.90–50.49)	52.98 (52.11–53.85)	56.38 (55.53–57.24)	55.69 (54.93–56.45)	55.64 (54.63–56.65)
Unknown	0.98 (0.91–1.04)	6.03 (5.63–6.44)	N/A	N/A	N/A	N/A

BMI = body mass index, CI = confidence interval, KNHANES = Korea National Health and Nutrition Examination Survey, WHtR = waist-to-height ratio.

† Central adiposity is divided into 2 groups: low-WHtR group (waist-to-height ratio < 0.50), and high-WHtR group (waist-to-height ratio ≥ 0.50).

‡ BMI was divided into 3 groups according to Asian-Pacific guidelines: underweight or normal weight (<23.00 kg/m^2^), overweight (23.00–24.90 kg/m^2^), and obese (25.00–34.90 kg/m^2^).

**Figure 1.**
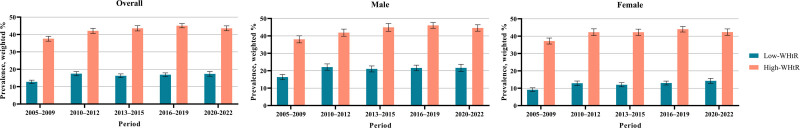
Nationwide trends in the prevalence of hypertension among Korean adults stratified by central adiposity groups from 2005 to 2022. WHtR = waist-to-height ratio.

Table 2 and Figure 1 show the prevalence of hypertension according to central adiposity groups from 2005 to 2022, as well as trends before and during the COVID-19 pandemic. Overall, the weighted prevalence of hypertension was 16.14% (95% CI: 15.64–16.64) in the low-WHtR group and 42.60% (41.97–43.23) in the high-WHtR group. In the low-WHtR group, males (20.47%; 95% CI: 19.69–21.25) had a higher prevalence of hypertension than females (12.33%; 11.78–12.88). Conversely, in the high-WHtR group, the prevalence of hypertension was relatively similar between males (43.44%; 42.57–44.31) and females (41.74%; 40.94–42.54).

**Table 2:** National trends in the prevalence of hypertension, stratified by central adiposity groups and β-coefficients of odds ratios before and during the COVID-19 pandemic (weighted % [95% CI]).

Variables		WHtR group†	Total	Pre-pandemic				During the pandemic	Trends before the pandemic, β (95% CI)	Trends in the pandemic, β (95% CI)	β_diff_ between 2005–2019 and 2019–2022 (95% CI)
				2005–2009	2010–2012	2013–2015	2016–2019	2020–2022			
Overall		Low-WHtR group	16.14 (15.64–16.64)	12.77 (11.79–13.76)	17.47 (16.28–18.67)	16.26 (15.18–17.34)	16.91 (15.89–17.94)	17.35 (16.00–18.71)	**1.12 (0.67–1.58**)	0.44 (−1.29 to 2.16)	−0.68 (−2.41 to 1.05)
		High-WHtR group	42.60 (41.97–43.23)	37.61 (36.18–39.04)	42.09 (40.57–43.60)	43.57 (42.07–45.06)	45.07 (43.86–46.27)	43.56 (42.16–44.96)	**2.33 (1.74–2.93**)	−1.51 (−3.39 to 0.38)	**−3.84 (−5.73 to −1.96**)
Sex											
Male		Low-WHtR group	20.47 (19.69–21.25)	16.35 (14.83–17.87)	22.12 (20.30–23.95)	21.06 (19.33–22.79)	21.60 (19.98–23.23)	21.65 (19.55–23.74)	**1.50 (0.79–2.22**)	0.04 (−2.62 to 2.71)	−1.46 (−4.13 to 1.21)
		High-WHtR group	43.44 (42.57–44.31)	38.05 (36.03–40.07)	41.89 (39.85–43.93)	44.93 (42.70–47.16)	46.07 (44.42–47.72)	44.57 (42.74–46.40)	**2.64 (1.81–3.47**)	−1.50 (−3.99 to 1.00)	**−4.14 (−6.64 to −1.65**)
Female		Low-WHtR group	12.33 (11.78–12.88)	9.16 (8.08–10.25)	12.89 (11.59–14.19)	12.04 (10.85–13.23)	13.01 (11.88–14.14)	14.26 (12.82–15.71)	**1.06 (0.56–1.56**)	1.25 (−0.60 to 3.11)	0.19 (−1.67 to 2.05)
		High-WHtR group	41.74 (40.94–42.54)	37.20 (35.46–38.94)	42.26 (40.31–44.21)	42.26 (40.44–44.08)	44.00 (42.40–45.60)	42.34 (40.50–44.18)	**2.00 (1.24–2.76**)	−1.66 (−4.12 to 0.80)	**−3.66 (−6.12 to −1.20**)
Age, yr											
Male	30–39	Low-WHtR group	7.95 (6.96–8.94)	8.75 (6.68–10.82)	8.20 (6.20–10.20)	8.31 (6.03–10.59)	6.42 (4.59–8.26)	7.62 (4.62–10.61)	−0.67 (−1.56 to 0.23)	1.19 (−2.32 to 4.71)	1.86 (−1.66 to 5.38)
		High-WHtR group	23.77 (21.92–25.61)	19.04 (15.37–22.71)	23.41 (19.05–27.77)	24.80 (20.21–29.39)	28.67 (24.88–32.46)	22.56 (18.43–26.68)	**3.03 (1.33–4.74**)	**−6.11 (−11.73 to −0.50**)	**−9.14 (−14.76 to −3.53**)
	40–49	Low-WHtR group	17.01 (15.60–18.42)	14.46 (11.86–17.06)	20.25 (16.66–23.84)	16.84 (13.80–19.88)	18.27 (15.26–21.27)	14.85 (11.28–18.41)	0.85 (−0.42 to 2.12)	−3.42 (−8.09 to 1.25)	−4.27 (−8.94 to 0.40)
		High-WHtR group	35.68 (33.96–37.41)	30.75 (27.10–34.40)	33.88 (29.76–37.99)	41.14 (36.62–45.67)	37.42 (34.23–40.61)	35.61 (31.79–39.43)	**2.58 (1.02–4.15**)	−1.81 (−6.78 to 3.16)	−4.39 (−9.36 to 0.58)
	50–59	Low-WHtR group	27.73 (25.84–29.63)	23.37 (19.41–27.32)	31.28 (26.67–35.89)	28.05 (23.87–32.23)	27.70 (23.99–31.41)	27.64 (22.94–32.34)	0.83 (−0.92 to 2.57)	−0.06 (−6.06 to 5.95)	−0.89 (−6.90 to 5.12)
		High-WHtR group	45.35 (43.69–47.02)	43.88 (40.02–47.74)	44.33 (40.36–48.31)	45.86 (41.90–49.82)	46.34 (43.13–49.56)	45.73 (42.08–49.38)	0.89 (−0.70 to 2.48)	−0.61 (−5.50 to 4.28)	−1.50 (−6.39 to 3.39)
	≥60	Low-WHtR group	38.97 (37.26–40.68)	30.45 (26.72–34.19)	42.63 (38.78–46.48)	40.75 (36.92–44.57)	42.40 (38.95–45.86)	38.20 (34.09–42.31)	**3.32 (1.71–4.93**)	−4.21 (−9.57 to 1.16)	**−7.53 (−12.90 to −2.17**)
		High-WHtR group	60.63 (59.38–61.88)	56.33 (53.06–59.61)	61.39 (58.52–64.25)	59.67 (56.46–62.87)	61.89 (59.61–64.18)	61.79 (59.24–64.33)	**1.42 (0.19–2.65**)	−0.11 (−3.51 to 3.30)	−1.53 (−4.94 to 1.88)
Female	30–39	Low-WHtR group	1.49 (1.15–1.83)	1.77 (0.98–2.57)	1.13 (0.51–1.75)	1.38 (0.68–2.08)	1.72 (0.98–2.46)	1.42 (0.49–2.35)	0.00 (−0.34 to 0.34)	−0.30 (−1.49 to 0.88)	−0.30 (−1.49 to 0.89)
		High-WHtR group	7.13 (5.98–8.29)	6.26 (4.12–8.40)	6.99 (4.29–9.69)	4.30 (2.14–6.46)	10.48 (7.63–13.33)	7.86 (4.83–10.88)	0.95 (−0.17 to 2.07)	−2.63 (−6.77 to 1.51)	−3.58 (−7.72 to 0.56)
	40–49	Low-WHtR group	6.64 (5.90–7.38)	6.14 (4.51–7.76)	6.70 (4.90–8.50)	7.13 (5.46–8.80)	5.74 (4.46–7.02)	7.50 (5.59–9.41)	−0.08 (−0.75 to 0.59)	1.76 (−0.54 to 4.05)	1.84 (−0.46 to 4.14)
		High-WHtR group	19.77 (18.25–21.29)	19.46 (16.41–22.51)	22.97 (19.16–26.78)	17.52 (13.91–21.13)	20.70 (17.58–23.83)	17.58 (14.19–20.97)	−0.12 (−1.53 to 1.28)	−3.12 (−7.72 to 1.48)	−3.00 (−7.60 to 1.60)
	50–59	Low-WHtR group	18.83 (17.46–20.19)	16.35 (12.92–19.77)	21.49 (18.14–24.83)	19.21 (16.10–22.31)	20.53 (17.87–23.20)	16.46 (13.59–19.33)	0.89 (−0.47 to 2.25)	**−4.07 (−7.99 to −0.15**)	**−4.96 (−8.88 to −1.04**)
		High-WHtR group	36.50 (35.01–38.00)	37.60 (34.03–41.17)	37.33 (33.96–40.70)	37.09 (33.72–40.46)	35.69 (32.90–38.48)	35.05 (31.39–38.71)	−0.61 (−2.04 to 0.82)	−0.64 (−5.24 to 3.96)	−0.03 (−4.63 to 4.57)
	≥60	Low-WHtR group	41.56 (39.72–43.40)	31.64 (28.07–35.20)	53.24 (48.42–58.07)	41.41 (36.98–45.84)	41.57 (37.91–45.22)	41.00 (37.16–44.83)	**1.69 (0.03–3.35**)	−0.57 (−5.87 to 4.73)	−2.26 (−7.56 to 3.04)
		High-WHtR group	62.79 (61.77–63.81)	58.63 (56.27–60.99)	66.11 (63.75–68.47)	64.58 (62.21–66.95)	63.67 (61.72–65.62)	60.83 (58.52–63.15)	**1.14 (0.17–2.11**)	−2.83 (−5.85 to 0.18)	**−3.97 (−6.99 to −0.96**)
Region of residence											
Male	Urban	Low-WHtR group	19.70 (18.85–20.56)	15.91 (14.17–17.65)	21.60 (19.58–23.62)	20.83 (18.93–22.73)	21.01 (19.24–22.77)	19.30 (17.09–21.51)	**1.47 (0.68–2.26**)	−1.71 (−4.54 to 1.13)	**−3.18 (−6.02 to −0.35**)
		High-WHtR group	43.30 (42.32–44.27)	37.75 (35.41–40.08)	42.22 (39.88–44.56)	45.14 (42.64–47.64)	45.40 (43.63–47.16)	44.23 (42.14–46.32)	**2.47 (1.55–3.40**)	−1.17 (−3.92 to 1.59)	**−3.64 (−6.40 to −0.89**)
	Rural	Low-WHtR group	23.94 (22.05–25.84)	18.17 (14.97–21.37)	24.19 (20.02–28.36)	22.02 (17.83–26.21)	25.23 (20.92–29.53)	33.26 (27.81–38.72)	**1.93 (0.23–3.62**)	**8.03 (1.10–14.96**)	6.10 (−0.83 to 13.03)
		High-WHtR group	44.01 (42.09–45.93)	39.12 (35.03–43.22)	40.82 (36.68–44.96)	44.11 (39.01–49.20)	49.21 (44.74–53.68)	46.13 (42.41–49.85)	**3.39 (1.46–5.32**)	−3.08 (−8.90 to 2.73)	**−6.47 (−12.29 to −0.66**)
Female	Urban	Low-WHtR group	11.50 (10.91–12.09)	8.30 (7.07–9.53)	11.82 (10.45–13.19)	11.65 (10.36–12.94)	12.32 (11.13–13.50)	12.96 (11.45–14.47)	**1.16 (0.62–1.71**)	0.64 (−1.29 to 2.58)	−0.52 (−2.46 to 1.42)
		High-WHtR group	40.36 (39.44–41.28)	35.35 (33.27–37.43)	40.77 (38.56–42.97)	41.26 (39.15–43.38)	42.41 (40.61–44.20)	41.14 (39.05–43.22)	**2.11 (1.23–2.98**)	−1.27 (−4.02 to 1.48)	**−3.38 (−6.13 to −0.63**)
	Rural	Low-WHtR group	17.43 (15.77–19.08)	13.25 (10.62–15.87)	18.83 (14.80–22.87)	14.53 (11.21–17.84)	17.90 (14.16–21.65)	23.70 (18.92–28.48)	1.03 (−0.42 to 2.48)	5.80 (−0.32 to 11.91)	4.77 (−1.35 to 10.89)
		High-WHtR group	46.61 (44.95–48.28)	42.96 (39.89–46.03)	46.29 (42.18–50.39)	45.86 (42.12–49.61)	50.56 (46.90–54.22)	47.66 (44.03–51.29)	**2.22 (0.68–3.76**)	−2.90 (−8.11 to 2.31)	−5.12 (−10.33 to 0.09)
BMI group‡											
Male	Underweight or normal weight	Low-WHtR group	20.27 (19.31–21.23)	17.92 (15.86–19.98)	20.51 (18.36–22.65)	20.53 (18.36–22.70)	21.92 (19.99–23.85)	20.42 (17.94–22.90)	**1.19 (0.29–2.09**)	−1.50 (−4.65 to 1.65)	−2.69 (−5.84 to 0.46)
		High-WHtR group	46.74 (44.15–49.34)	41.10 (35.34–46.87)	43.40 (37.38–49.42)	48.15 (41.76–54.55)	50.12 (45.24–55.00)	49.12 (43.31–54.92)	**3.17 (0.76–5.58**)	−1.00 (−8.56 to 6.55)	−4.17 (−11.73 to 3.39)
	Overweight	Low-WHtR group	20.89 (19.39–22.40)	18.79 (15.60–21.99)	22.95 (19.56–26.35)	20.49 (17.18–23.79)	20.56 (17.45–23.68)	21.62 (17.70–25.53)	0.25 (−1.17 to 1.67)	1.05 (−3.95 to 6.06)	0.80 (−4.21 to 5.81)
		High-WHtR group	39.03 (37.48–40.59)	35.61 (32.14–39.08)	39.80 (36.09–43.51)	38.69 (34.81–42.57)	41.54 (38.60–44.48)	38.79 (35.42–42.17)	**1.67 (0.22–3.12**)	−2.75 (−7.25 to 1.76)	−4.42 (−8.93 to 0.09)
	Obese	Low-WHtR group	24.75 (21.88–27.63)	23.87 (17.50–30.25)	31.64 (24.69–38.59)	24.89 (19.05–30.74)	20.55 (15.40–25.70)	20.58 (13.12–28.04)	−1.90 (−4.55 to 0.75)	0.04 (−9.01 to 9.09)	1.94 (−7.11 to 10.99)
		High-WHtR group	44.71 (43.64–45.79)	38.66 (36.18–41.14)	42.49 (39.94–45.03)	46.91 (44.23–49.58)	47.29 (45.34–49.24)	46.19 (43.80–48.58)	**2.94 (1.94–3.94**)	−1.10 (−4.20 to 2.00)	**−4.04 (−7.14 to −0.94**)
Female	Underweight or normal weight	Low-WHtR group	11.71 (11.12–12.30)	9.57 (8.38–10.76)	12.94 (11.50–14.38)	11.64 (10.36–12.92)	12.40 (11.22–13.59)	11.65 (10.20–13.10)	**0.66 (0.12–1.21**)	−0.76 (−2.64 to 1.12)	−1.42 (−3.30 to 0.46)
		High-WHtR group	41.13 (39.35–42.91)	31.35 (27.80–34.91)	44.73 (40.35–49.11)	44.14 (39.74–48.54)	43.09 (39.58–46.60)	42.15 (38.07–46.24)	**3.35 (1.72–4.98**)	−0.93 (−6.30 to 4.43)	−4.28 (−9.65 to 1.09)
	Overweight	Low-WHtR group	13.42 (11.99–14.84)	12.44 (9.14–15.73)	12.50 (9.59–15.40)	12.93 (9.97–15.90)	14.08 (11.19–16.98)	15.55 (11.53–19.57)	0.54 (−0.84 to 1.92)	1.47 (−3.47 to 6.41)	0.93 (−4.01 to 5.87)
		High-WHtR group	37.46 (36.09–38.83)	32.69 (29.90–35.48)	39.76 (36.58–42.94)	36.97 (33.76–40.18)	40.02 (37.25–42.80)	37.44 (34.16–40.72)	**1.90 (0.64–3.16**)	−2.58 (−6.93 to 1.77)	**−4.48 (−8.83 to −0.13**)
	Obese	Low-WHtR group	16.48 (12.79–20.17)	13.85 (6.17–21.54)	13.36 (7.33–19.39)	18.60 (9.63–27.57)	13.80 (7.33–20.26)	21.30 (11.58–31.01)	0.53 (−2.65 to 3.70)	7.50 (−4.17 to 19.17)	6.97 (−4.70 to 18.64)
		High-WHtR group	44.08 (43.04–45.11)	41.43 (39.14–43.73)	42.81 (40.37–45.25)	44.44 (42.10–46.77)	46.26 (44.23–48.28)	44.90 (42.47–47.33)	**1.62 (0.64–2.59**)	−1.36 (−4.53 to 1.81)	−2.98 (−6.15 to 0.19)
Level of education											
Male	Middle school or lower education	Low-WHtR group	34.17 (32.27–36.07)	24.22 (21.01–27.43)	37.53 (33.35–41.71)	33.53 (29.25–37.81)	39.38 (35.03–43.74)	46.57 (40.52–52.62)	**4.46 (2.76–6.16**)	7.19 (−0.26 to 14.64)	2.73 (−4.72 to 10.18)
		High-WHtR group	55.25 (53.70–56.81)	47.29 (43.97–50.60)	50.89 (47.38–54.40)	54.96 (51.16–58.76)	61.26 (58.31–64.22)	63.88 (60.25–67.50)	**4.59 (3.16–6.02**)	2.61 (−2.05 to 7.28)	−1.98 (−6.65 to 2.69)
	High school or higher education	Low-WHtR group	17.58 (16.76–18.40)	14.00 (12.38–15.61)	18.28 (16.35–20.21)	18.33 (16.45–20.20)	18.85 (17.15–20.54)	18.51 (16.39–20.62)	**1.45 (0.70–2.20**)	−0.34 (−3.05 to 2.37)	−1.79 (−4.50 to 0.92)
		High-WHtR group	39.69 (38.69–40.69)	33.64 (31.26–36.03)	38.12 (35.62–40.62)	41.40 (38.82–43.98)	41.94 (40.09–43.80)	40.79 (38.74–42.83)	**2.69 (1.73–3.64**)	−1.16 (−3.92 to 1.61)	**−3.85 (−6.62 to −1.09**)
Female	Middle school or lower education	Low-WHtR group	32.23 (30.61–33.86)	21.15 (18.54–23.77)	35.48 (31.96–38.99)	31.52 (27.62–35.42)	37.93 (34.15–41.70)	42.30 (37.44–47.16)	**4.93 (3.49–6.37**)	4.37 (−1.78 to 10.52)	−0.56 (−6.71 to 5.59)
		High-WHtR group	56.64 (55.62–57.65)	47.91 (45.75–50.08)	56.57 (54.25–58.89)	58.31 (56.05–60.57)	60.35 (58.29–62.41)	61.61 (59.14–64.08)	**3.93 (2.98–4.88**)	1.26 (−1.95 to 4.46)	−2.67 (−5.88 to 0.54)
	High school or higher education	Low-WHtR group	7.92 (7.42–8.43)	4.99 (4.05–5.92)	6.18 (5.15–7.22)	8.07 (6.94–9.19)	9.00 (8.01–10.00)	10.34 (8.97–11.71)	**1.39 (0.95–1.83**)	1.34 (−0.36 to 3.03)	−0.05 (−1.75 to 1.65)
		High-WHtR group	25.54 (24.51–26.56)	19.39 (17.06–21.73)	22.09 (19.77–24.41)	22.63 (20.30–24.96)	28.87 (26.90–30.85)	29.77 (27.51–32.03)	**3.04 (2.07–4.01**)	0.89 (−2.11 to 3.89)	−2.15 (−5.15 to 0.85)
Household income											
Male	Lowest and second quartile	Low-WHtR group	25.27 (23.99–26.56)	20.26 (17.91–22.62)	23.88 (21.06–26.70)	25.93 (23.03–28.83)	27.88 (25.03–30.72)	30.98 (27.26–34.69)	**2.50 (1.33–3.68**)	3.10 (−1.61 to 7.81)	0.60 (−4.11 to 5.31)
		High-WHtR group	50.16 (48.83–51.49)	43.33 (40.41–46.26)	48.65 (45.40–51.90)	50.93 (47.61–54.25)	53.63 (51.17–56.09)	52.45 (49.52–55.39)	**3.27 (2.05–4.50**)	−1.18 (−5.01 to 2.66)	**−4.45 (−8.29 to −0.62**)
	Third and highest quartile	Low-WHtR group	17.73 (16.77–18.68)	14.01 (12.19–15.83)	20.86 (18.43–23.29)	18.33 (16.15–20.51)	18.39 (16.52–20.26)	17.36 (14.91–19.81)	**1.11 (0.27–1.95**)	−1.03 (−4.12 to 2.05)	−2.14 (−5.23 to 0.95)
		High-WHtR group	39.17 (38.07–40.27)	34.36 (31.76–36.95)	37.10 (34.43–39.76)	40.99 (38.13–43.84)	41.30 (39.31–43.29)	40.28 (37.96–42.60)	**2.40 (1.36–3.43**)	−1.02 (−4.08 to 2.04)	**−3.42 (−6.48 to −0.36**)
Female	Lowest and second quartile	Low-WHtR group	18.00 (16.96–19.04)	13.21 (11.41–15.02)	18.52 (15.96–21.07)	18.25 (15.97–20.53)	18.47 (16.41–20.53)	21.73 (18.93–24.53)	**1.53 (0.64–2.43**)	3.26 (−0.25 to 6.76)	1.73 (−1.78 to 5.24)
		High-WHtR group	49.09 (48.06–50.11)	43.32 (41.21–45.42)	48.61 (46.10–51.13)	49.31 (46.92–51.70)	53.00 (51.04–54.97)	50.34 (47.91–52.77)	**2.95 (2.02–3.88**)	−2.66 (−5.80 to 0.48)	**−5.61 (−8.75 to −2.47**)
	Third and highest quartile	Low-WHtR group	9.41 (8.80–10.02)	6.87 (5.62–8.12)	9.25 (7.95–10.55)	9.09 (7.75–10.42)	10.38 (9.12–11.63)	11.00 (9.43–12.57)	**1.03 (0.47–1.60**)	0.62 (−1.39 to 2.64)	−0.41 (−2.43 to 1.61)
		High-WHtR group	33.07 (31.98–34.16)	29.86 (27.42–32.31)	34.08 (31.59–36.56)	33.43 (30.80–36.06)	33.33 (31.20–35.46)	34.20 (31.71–36.68)	0.92 (−0.11 to 1.96)	0.87 (−2.41 to 4.15)	−0.05 (−3.33 to 3.23)
Smoking status											
Male	Smoker or ex-smoker	Low-WHtR group	21.55 (20.64–22.45)	18.14 (16.32–19.96)	22.62 (20.58–24.67)	21.64 (19.60–23.68)	22.29 (20.49–24.10)	23.25 (20.74–25.75)	**1.14 (0.31–1.96**)	0.95 (−2.14 to 4.05)	−0.19 (−3.29 to 2.91)
		High-WHtR group	44.15 (43.20–45.09)	37.81 (35.69–39.93)	43.17 (40.94–45.41)	45.18 (42.65–47.71)	46.54 (44.75–48.34)	46.26 (44.31–48.21)	**2.74 (1.85–3.63**)	−0.29 (−2.96 to 2.38)	**−3.03 (−5.70 to −0.36**)
	Nonsmoker	Low-WHtR group	18.78 (17.16–20.40)	20.16 (16.08–24.23)	19.61 (15.68–23.53)	18.99 (15.54–22.44)	19.16 (15.93–22.38)	16.31 (12.68–19.94)	−0.35 (−1.98 to 1.28)	−2.85 (−7.71 to 2.01)	−2.50 (−7.36 to 2.36)
		High-WHtR group	40.28 (38.34–42.22)	39.33 (34.62–44.03)	34.41 (29.59–39.23)	43.80 (39.25–48.36)	44.14 (40.37–47.92)	38.32 (34.43–42.21)	**2.44 (0.53–4.36**)	**−5.82 (−11.29 to −0.36**)	**−8.26 (−13.73 to −2.80**)
Female	Smoker or ex-smoker	Low-WHtR group	10.88 (9.46–12.30)	10.61 (7.06–14.16)	11.34 (8.19–14.50)	7.99 (5.05–10.93)	12.83 (9.85–15.80)	11.10 (7.88–14.33)	0.42 (−1.03 to 1.86)	−1.72 (−6.12 to 2.67)	−2.14 (−6.54 to 2.26)
		High-WHtR group	34.68 (32.39–36.97)	39.68 (34.52–44.83)	33.29 (27.97–38.61)	36.12 (30.57–41.68)	35.87 (31.25–40.49)	29.47 (24.68–34.26)	−0.83 (−3.04 to 1.38)	−6.40 (−13.03 to 0.24)	−5.57 (−12.21 to 1.07)
	Nonsmoker	Low-WHtR group	12.86 (12.26–13.46)	10.38 (9.14–11.62)	13.12 (11.73–14.51)	12.53 (11.29–13.77)	13.04 (11.84–14.23)	14.72 (13.14–16.31)	**0.70 (0.15–1.25**)	1.69 (−0.32 to 3.69)	0.99 (−1.02 to 3.00)
		High-WHtR group	42.54 (41.70–43.37)	36.89 (35.12–38.65)	43.34 (41.30–45.38)	42.91 (41.05–44.77)	44.85 (43.19–46.51)	43.82 (41.86–45.78)	**2.29 (1.51–3.06**)	−1.03 (−3.63 to 1.57)	**−3.32 (−5.92 to −0.72**)

Beta values were multiplied by 100 due to their small magnitudes. The values in bold font represent significant variance (*P* < .05).

BMI = body mass index, CI = confidence interval, KNHANES = Korea National Health and Nutrition Examination Survey, WHtR = waist-to-height ratio.

† Central adiposity is divided into 2 groups: low-WHtR group (waist-to-height ratio < 0.50), and high-WHtR group (waist-to-height ratio ≥ 0.50).

‡ BMI was divided into 3 groups according to Asian-Pacific guidelines: underweight or normal weight (<23.00 kg/m^2^), overweight (23.00–24.90 kg/m^2^), and obese (25.00–34.90 kg/m^2^).

During the pre-pandemic period, the proportion of individuals with hypertension in the low-WHtR group increased from 12.77% (11.79–13.76) in 2005 to 2009 to 16.91% (15.89–17.94) in 2016 to 2019 (β = 1.12; 95% CI: 0.67–1.58). Among those in the high-WHtR group, the prevalence increased steadily from 37.61% (36.18–39.04) in 2005 to 2009 to 45.07% (43.86–46.27) in 2016 to 2019 (β = 2.33; 1.74–2.93), indicating a more rapid increase than that observed in the low-WHtR group.

Table 3 presents the influence of covariates on hypertension prevalence. In the full dataset, the older population and higher BMI were confirmed as significant risk factors for hypertension across both central adiposity groups. Notably, the magnitude of the aOR for hypertension by age group was greater in the low-WHtR group compared to the high-WHtR group. A similar pattern was observed for BMI.

**Table 3:** Adjusted odds ratios (95% CI) for hypertension prevalence, stratified by central adiposity groups and subgroups of covariates before and during COVID-19.

Variables		Overall (2005–2022)		Before the pandemic (2005–2019)		During the pandemic (2020–2022)		Ratio of OR (95% CI), during the pandemic compared to before the pandemic (reference)	
		Male	Female	Male	Female	Male	Female	Male	Female
		Adjusted OR (95% CI)	Adjusted OR (95% CI)	Adjusted OR (95% CI)	Adjusted OR (95% CI)	Adjusted OR (95% CI)	Adjusted OR (95% CI)	Ratio of OR (95% CI)	Ratio of OR (95% CI)
Low-WHtR group†									
Age, yr	30–39	1.00 (ref)	1.00 (ref)	1.00 (ref)	1.00 (ref)	1.00 (ref)	1.00 (ref)	1.00 (ref)	1.00 (ref)
	40–49	**2.41 (2.04–2.86**)	**4.58 (3.56–5.89**)	**2.49 (2.09–2.97**)	**4.35 (3.33–5.69**)	**2.02 (1.20–3.39**)	**5.73 (2.82–11.66**)	0.81 (0.47–1.40)	1.32 (0.62–2.81)
	50–59	**4.36 (3.68–5.17**)	**13.97 (10.94–17.83**)	**4.45 (3.72–5.32**)	**14.11 (10.82–18.40**)	**4.13 (2.51–6.80**)	**14.20 (7.30–27.61**)	0.93 (0.55–1.58)	1.01 (0.49–2.06)
	≥60	**6.83 (5.78–8.08**)	**34.67 (27.14–44.29**)	**7.36 (6.16–8.79**)	**36.36 (27.74–47.66**)	**5.10 (3.12–8.34**)	**33.21 (17.50–63.02**)	0.69 (0.41–1.17)	0.91 (0.46–1.83)
Region of residence	Urban	1.00 (ref)	1.00 (ref)	1.00 (ref)	1.00 (ref)	1.00 (ref)	1.00 (ref)	1.00 (ref)	1.00 (ref)
	Rural	0.99 (0.88–1.12)	1.07 (0.95–1.22)	0.90 (0.78–1.03)	0.97 (0.85–1.12)	**1.60 (1.20–2.13**)	**1.51 (1.15–1.99**)	**1.78 (1.29–2.45**)	**1.56 (1.15–2.12**)
BMI group‡	Underweight or normal weight	1.00 (ref)	1.00 (ref)	1.00 (ref)	1.00 (ref)	1.00 (ref)	1.00 (ref)	1.00 (ref)	1.00 (ref)
	Overweight	**1.55 (1.37–1.74**)	**1.46 (1.25–1.70**)	**1.49 (1.31–1.70**)	**1.35 (1.15–1.59**)	**1.78 (1.32–2.42**)	**1.93 (1.32–2.83**)	1.19 (0.86–1.66)	1.43 (0.94–2.16)
	Obese	**2.39 (2.00–2.85**)	**2.21 (1.65–2.96**)	**2.41 (1.99–2.91**)	**1.96 (1.41–2.73**)	**2.02 (1.21–3.36**)	**3.15 (1.73–5.71**)	0.84 (0.49–1.45)	1.61 (0.81–3.18)
Education level	High school or higher education	1.00 (ref)	1.00 (ref)	1.00 (ref)	1.00 (ref)	1.00 (ref)	1.00 (ref)	1.00 (ref)	1.00 (ref)
	Middle school or lower education	1.12 (0.99–1.27)	**1.53 (1.35–1.74**)	1.06 (0.92–1.21)	**1.55 (1.35–1.78**)	**1.44 (1.01–2.07**)	1.31 (0.93–1.84)	1.36 (0.93–1.99)	0.85 (0.58–1.22)
Household income	Third and highest quartile	1.00 (ref)	1.00 (ref)	1.00 (ref)	1.00 (ref)	1.00 (ref)	1.00 (ref)	1.00 (ref)	1.00 (ref)
	Lowest and second quartile	**1.13 (1.01–1.25**)	1.09 (0.97–1.22)	1.08 (0.97–1.21)	1.06 (0.93–1.21)	1.31 (1.00–1.73)	1.16 (0.90–1.50)	1.21 (0.90–1.63)	1.09 (0.82–1.46)
Smoking status	Nonsmoker	1.00 (ref)	1.00 (ref)	1.00 (ref)	1.00 (ref)	1.00 (ref)	1.00 (ref)	1.00 (ref)	1.00 (ref)
	Smoker or ex-smoker	1.10 (0.97–1.25)	**1.24 (1.04–1.47**)	1.05 (0.92–1.20)	**1.23 (1.01–1.49**)	**1.45 (1.06–1.99**)	1.27 (0.85–1.90)	1.38 (0.98–1.94)	1.03 (0.66–1.61)
High-WHtR group†									
Age, yr	30–39	1.00 (ref)	1.00 (ref)	1.00 (ref)	1.00 (ref)	1.00 (ref)	1.00 (ref)	1.00 (ref)	1.00 (ref)
	40–49	**1.74 (1.54–1.97**)	**2.99 (2.46–3.64**)	**1.72 (1.50–1.97**)	**3.13 (2.52–3.88**)	**1.92 (1.43–2.56**)	**2.54 (1.59–4.07**)	1.12 (0.81–1.54)	0.81 (0.48–1.36)
	50–59	**2.57 (2.28–2.91**)	**6.19 (5.12–7.48**)	**2.54 (2.21–2.91**)	**6.30 (5.12–7.75**)	**3.09 (2.33–4.10**)	**6.47 (4.11–10.19**)	1.22 (0.89–1.67)	1.03 (0.62–1.69)
	≥60	**4.50 (3.99–5.08**)	**15.41 (12.73–18.64**)	**4.43 (3.86–5.08**)	**16.14 (13.09–19.89**)	**5.83 (4.39–7.75**)	**15.42 (9.89–24.03**)	1.32 (0.96–1.80)	0.96 (0.58–1.56)
Region of residence	Urban	1.00 (ref)	1.00 (ref)	1.00 (ref)	1.00 (ref)	1.00 (ref)	1.00 (ref)	1.00 (ref)	1.00 (ref)
	Rural	**0.89 (0.82–0.97**)	0.99 (0.92–1.08)	**0.88 (0.79–0.98**)	1.01 (0.92–1.10)	0.92 (0.78–1.10)	0.93 (0.77–1.13)	1.05 (0.85–1.28)	0.92 (0.75–1.14)
BMI group	Underweight or normal weight	1.00 (ref)	1.00 (ref)	1.00 (ref)	1.00 (ref)	1.00 (ref)	1.00 (ref)	1.00 (ref)	1.00 (ref)
	Overweight	1.01 (0.89–1.14)	**1.12 (1.02–1.23**)	1.03 (0.89–1.19)	**1.11 (1.00–1.24**)	0.93 (0.70–1.25)	1.16 (0.91–1.47)	0.90 (0.65–1.25)	1.05 (0.80–1.36)
	Obese	**1.88 (1.66–2.13**)	**1.89 (1.73–2.06**)	**1.86 (1.62–2.13**)	**1.84 (1.67–2.03**)	**1.93 (1.45–2.57**)	**2.11 (1.71–2.59**)	1.04 (0.76–1.42)	1.15 (0.91–1.44)
Education level	High school or higher education	1.00 (ref)	1.00 (ref)	1.00 (ref)	1.00 (ref)	1.00 (ref)	1.00 (ref)	1.00 (ref)	1.00 (ref)
	Middle school or lower education	1.05 (0.96–1.15)	**1.47 (1.36–1.59**)	1.00 (0.90–1.10)	**1.43 (1.31–1.57**)	**1.40 (1.13–1.74**)	**1.60 (1.34–1.92**)	**1.40 (1.10–1.78**)	1.12 (0.91–1.37)
Household income	Third and highest quartile	1.00 (ref)	1.00 (ref)	1.00 (ref)	1.00 (ref)	1.00 (ref)	1.00 (ref)	1.00 (ref)	1.00 (ref)
	Lowest and second quartile	**1.22 (1.13–1.32**)	**1.15 (1.07–1.23**)	**1.23 (1.13–1.34**)	**1.15 (1.07–1.24**)	**1.24 (1.04–1.48**)	1.15 (0.97–1.36)	1.01 (0.83–1.23)	1.00 (0.83–1.20)
Smoking status	Nonsmoker	1.00 (ref)	1.00 (ref)	1.00 (ref)	1.00 (ref)	1.00 (ref)	1.00 (ref)	1.00 (ref)	1.00 (ref)
	Smoker or ex-smoker	1.09 (1.00–1.20)	1.03 (0.91–1.16)	1.06 (0.96–1.18)	1.06 (0.92–1.21)	**1.21 (1.00–1.45**)	0.91 (0.69–1.19)	1.14 (0.92–1.41)	0.86 (0.63–1.16)

Adjusted for age, region, educational background, household income, and smoking status. The values in bold font represent a significant variance (*P* < .05).

BMI = body mass index, CI = confidence interval, KNHANES = Korea National Health and Nutrition Examination Survey, OR = odds ratio, WHtR = waist-to-height ratio.

† Central adiposity is divided into 2 groups: low-WHtR group (waist-to-height ratio < 0.50), and high-WHtR group (waist-to-height ratio ≥ 0.50).

‡ BMI was divided into 3 groups according to Asian-Pacific guidelines: underweight or normal weight (<23.00 kg/m^2^), overweight (23.00–24.90 kg/m^2^), and obese (25.00–34.90 kg/m^2^).

Table 4 illustrates the effect of central adiposity groups on hypertension prevalence within subgroups. Using the low-WHtR group as the reference, the aOR for hypertension was higher in females aged 30 to 59 years in the high-WHtR group; however, among individuals aged 60 years or older, the aOR was higher in males. Additionally, the odds of hypertension were significantly higher in the high-WHtR group in all subgroup analyses, including those stratified by socioeconomic and behavioral factors.

**Table 4:** Adjusted odds ratios (95% CI) for hypertension prevalence between central adiposity groups before and during COVID-19.

Variables		Overall (2005–2022)		Before the pandemic (2005–2019)		During the pandemic (2020–2022)		Ratio of OR (95% CI), during the pandemic compared to before the pandemic (reference)	
		Male	Female	Male	Female	Male	Female	Male	Female
		Adjusted OR (95% CI)	Adjusted OR (95% CI)	Adjusted OR (95% CI)	Adjusted OR (95% CI)	Adjusted OR (95% CI)	Adjusted OR (95% CI)	Ratio of OR (95% CI)	Ratio of OR (95% CI)
Overall	Low-WHtR group†	1.00 (ref)	1.00 (ref)	1.00 (ref)	1.00 (ref)	1.00 (ref)	1.00 (ref)	1.00 (ref)	1.00 (ref)
	High-WHtR group†	**2.53 (2.38–2.69**)	**2.50 (2.34–2.67**)	**2.48 (2.32–2.65**)	**2.45 (2.28–2.64**)	**2.81 (2.41–3.26**)	**2.71 (2.33–3.14**)	1.13 (0.96–1.34)	1.11 (0.94–1.31)
Age, yr									
30–39	Low-WHtR group	1.00 (ref)	1.00 (ref)	1.00 (ref)	1.00 (ref)	1.00 (ref)	1.00 (ref)	1.00 (ref)	1.00 (ref)
	High-WHtR group	**3.68 (3.10–4.36**)	**5.04 (3.76–6.74**)	**3.74 (3.11–4.49**)	**4.76 (3.48–6.51**)	**3.62 (2.21–5.92**)	**6.66 (2.97–14.94**)	0.97 (0.57–1.64)	1.40 (0.59–3.33)
40–49	Low-WHtR group	1.00 (ref)	1.00 (ref)	1.00 (ref)	1.00 (ref)	1.00 (ref)	1.00 (ref)	1.00 (ref)	1.00 (ref)
	High-WHtR group	**2.63 (2.32–2.98**)	**3.29 (2.83–3.84**)	**2.55 (2.23–2.92**)	**3.46 (2.92–4.10**)	**3.14 (2.24–4.40**)	**2.70 (1.88–3.88**)	1.23 (0.86–1.77)	0.78 (0.52–1.16)
50–59	Low-WHtR group	1.00 (ref)	1.00 (ref)	1.00 (ref)	1.00 (ref)	1.00 (ref)	1.00 (ref)	1.00 (ref)	1.00 (ref)
	High-WHtR group	**2.11 (1.88–2.38**)	**2.26 (2.02–2.53**)	**2.08 (1.83–2.37**)	**2.16 (1.91–2.44**)	**2.27 (1.71–3.03**)	**2.67 (2.04–3.49**)	1.09 (0.80–1.49)	1.24 (0.92–1.66)
≥60	Low-WHtR group	1.00 (ref)	1.00 (ref)	1.00 (ref)	1.00 (ref)	1.00 (ref)	1.00 (ref)	1.00 (ref)	1.00 (ref)
	High-WHtR group	**2.37 (2.16–2.59**)	**2.15 (1.96–2.36**)	**2.23 (2.02–2.47**)	**2.05 (1.85–2.27**)	**2.85 (2.30–3.54**)	**2.51 (2.05–3.07**)	**1.28 (1.01–1.62**)	1.22 (0.98–1.54)
Region of residence									
Urban	Low-WHtR group	1.00 (ref)	1.00 (ref)	1.00 (ref)	1.00 (ref)	1.00 (ref)	1.00 (ref)	1.00 (ref)	1.00 (ref)
	High-WHtR group	**2.60 (2.43–2.79**)	**2.56 (2.38–2.76**)	**2.51 (2.33–2.70**)	**2.47 (2.27–2.68**)	**3.14 (2.64–3.73**)	**2.92 (2.47–3.46**)	**1.25 (1.04–1.51**)	1.18 (0.98–1.43)
Rural	Low-WHtR group	1.00 (ref)	1.00 (ref)	1.00 (ref)	1.00 (ref)	1.00 (ref)	1.00 (ref)	1.00 (ref)	1.00 (ref)
	High-WHtR group	**2.24 (1.97–2.54**)	**2.26 (1.99–2.57**)	**2.35 (2.03–2.72**)	**2.39 (2.07–2.76**)	**1.76 (1.33–2.32**)	**1.78 (1.33–2.40**)	0.75 (0.55–1.03)	0.74 (0.54–1.03)
BMI group‡									
Underweight or normal weight	Low-WHtR group	1.00 (ref)	1.00 (ref)	1.00 (ref)	1.00 (ref)	1.00 (ref)	1.00 (ref)	1.00 (ref)	1.00 (ref)
	High-WHtR group	**1.82 (1.59–2.07**)	**1.61 (1.44–1.79**)	**1.73 (1.49–2.00**)	**1.57 (1.40–1.77**)	**2.20 (1.64–2.94**)	**1.75 (1.36–2.24**)	1.27 (0.92–1.76)	1.11 (0.85–1.47)
Overweight	Low-WHtR group	1.00 (ref)	1.00 (ref)	1.00 (ref)	1.00 (ref)	1.00 (ref)	1.00 (ref)	1.00 (ref)	1.00 (ref)
	High-WHtR group	**1.26 (1.11–1.44**)	**1.47 (1.26–1.71**)	**1.31 (1.14–1.51**)	**1.56 (1.31–1.84**)	**1.13 (0.82–1.55**)	1.13 (0.77–1.66)	0.86 (0.61–1.22)	0.72 (0.48–1.10)
Obese	Low-WHtR group	1.00 (ref)	1.00 (ref)	1.00 (ref)	1.00 (ref)	1.00 (ref)	1.00 (ref)	1.00 (ref)	1.00 (ref)
	High-WHtR group	**1.75 (1.49–2.06**)	**1.79 (1.36–2.35**)	**1.69 (1.42–2.01**)	**1.94 (1.43–2.65**)	**2.24 (1.46–3.42**)	1.44 (0.81–2.54)	1.33 (0.84–2.10)	0.74 (0.39–1.42)
Education level									
Middle school or lower education	Low-WHtR group	1.00 (ref)	1.00 (ref)	1.00 (ref)	1.00 (ref)	1.00 (ref)	1.00 (ref)	1.00 (ref)	1.00 (ref)
	High-WHtR group	**2.17 (1.94–2.42**)	**2.18 (1.99–2.38**)	**2.14 (1.90–2.40**)	**2.11 (1.92–2.33**)	**2.16 (1.57–2.97**)	**2.63 (2.05–3.38**)	1.01 (0.72–1.42)	1.25 (0.95–1.63)
High school or higher education	Low-WHtR group	1.00 (ref)	1.00 (ref)	1.00 (ref)	1.00 (ref)	1.00 (ref)	1.00 (ref)	1.00 (ref)	1.00 (ref)
	High-WHtR group	**2.65 (2.47–2.85**)	**2.78 (2.54–3.04**)	**2.60 (2.40–2.82**)	**2.80 (2.53–3.11**)	**2.93 (2.49–3.46**)	**2.73 (2.26–3.29**)	1.13 (0.94–1.35)	0.98 (0.79–1.21)
Household income									
Lowest and second quartile	Low-WHtR group	1.00 (ref)	1.00 (ref)	1.00 (ref)	1.00 (ref)	1.00 (ref)	1.00 (ref)	1.00 (ref)	1.00 (ref)
	High-WHtR group	**2.52 (2.30–2.76**)	**2.39 (2.18–2.62**)	**2.52 (2.28–2.79**)	**2.36 (2.14–2.61**)	**2.50 (2.00–3.13**)	**2.56 (2.06–3.18**)	0.99 (0.78–1.27)	1.08 (0.85–1.38)
Third and highest quartile	Low-WHtR group	1.00 (ref)	1.00 (ref)	1.00 (ref)	1.00 (ref)	1.00 (ref)	1.00 (ref)	1.00 (ref)	1.00 (ref)
	High-WHtR group	**2.53 (2.33–2.75**)	**2.60 (2.37–2.86**)	**2.44 (2.23–2.67**)	**2.54 (2.29–2.83**)	**2.99 (2.44–3.66**)	**2.81 (2.28–3.46**)	1.23 (0.98–1.53)	1.11 (0.88–1.40)
Smoking status									
Smoker or ex-smoker	Low-WHtR group	1.00 (ref)	1.00 (ref)	1.00 (ref)	1.00 (ref)	1.00 (ref)	1.00 (ref)	1.00 (ref)	1.00 (ref)
	High-WHtR group	**2.53 (2.36–2.71**)	**2.35 (1.93–2.87**)	**2.48 (2.31–2.67**)	**2.38 (1.90–2.96**)	**2.69 (2.27–3.19**)	**2.27 (1.45–3.56**)	1.08 (0.90–1.30)	0.95 (0.58–1.57)
Nonsmoker	Low-WHtR group	1.00 (ref)	1.00 (ref)	1.00 (ref)	1.00 (ref)	1.00 (ref)	1.00 (ref)	1.00 (ref)	1.00 (ref)
	High-WHtR group	**2.56 (2.22–2.94**)	**2.52 (2.35–2.70**)	**2.45 (2.10–2.86**)	**2.46 (2.28–2.66**)	**3.57 (2.53–5.04**)	**2.75 (2.33–3.24**)	1.46 (1.00–2.13)	1.12 (0.93–1.34)

Adjusted for age, region, educational background, household income, and smoking status. The values in bold font represent a significant variance (*P* < .05).

BMI = body mass index, CI = confidence interval, KNHANES = Korea National Health and Nutrition Examination Survey, OR = odds ratio, WHtR = waist-to-height ratio.

† Central adiposity is divided into 2 groups: low-WHtR group (waist-to-height ratio < 0.50), and high-WHtR group (waist-to-height ratio ≥ 0.50).

‡ BMI was divided into 3 groups according to Asian-Pacific guidelines: underweight or normal weight (<23.00 kg/m^2^), overweight (23.00–24.90 kg/m^2^), and obese (25.00–34.90 kg/m^2^).

## 4. Discussion

### 4.1. Key findings

This study provides a comprehensive analysis of hypertension trends across central adiposity groups and associated risk factors, including age and BMI, encompassing 98,396 adults in South Korea. From 2005 to 2019, hypertension prevalence increased in both central adiposity groups, with a more pronounced increase in the high-WHtR group compared with the low-WHtR group. In the low-WHtR group, males exhibited significantly higher prevalence of hypertension compared to females (20.47% in males vs 12.33% in females), whereas, in the high-WHtR group, prevalence rates were similarly high for both sexes (43.44% vs 41.74%). Subgroup analyses further identified that among individuals aged 30 to 59 years, females in the high-WHtR group had a higher odds ratio for hypertension compared to males. However, among those aged 60 years and older, males exhibited a greater odds ratio compared to females. Although socioeconomic and behavioral factors had a slight influence on the results, their impact remained consistent and did not diminish the strong association between central adiposity and hypertension risk.

### 4.2. Comparison with previous studies

Some previous studies have explored the trends in hypertension prevalence and its association with socioeconomic and behavioral factors in South Korea.^[21]^ However, few studies have utilized WHtR to classify groups and analyze the trends in hypertension prevalence and its relationship with these factors. WHtR has been suggested as a more reliable marker of cardiometabolic risk factors than waist circumference or BMI in previous studies,^[5]^ and WHtR has also been reported to predict hypertension more accurately than BMI in the Korean Genome and Epidemiology Study.^[6,22]^ Given the accumulating evidence, evaluating long-term trends in hypertension prevalence using WHtR as a key metric offers meaningful clinical and public health insights.^[6,22]^ In addition, most previous studies have not sufficiently addressed sex differences in these trends and associations.^[11]^ This may overlook the significant influence of hormonal factors and physiological mechanisms that differ between the sexes on the occurrence of cardiovascular disease.^[23]^

This study differs from previous studies by emphasizing variations in the relationship between central obesity and hypertension across sexes. In particular, we emphasized the interaction between central obesity, sex and age-specific trends to better capture the variation in the prevalence of hypertension within subgroups. This comprehensive approach contributes to a deeper understanding of how specific risk factors interact with age and sex to influence the risk of hypertension.

### 4.3. Plausible underlying mechanisms

Estrogen in females exerts multifaceted regulatory effects on adipose tissue distribution and metabolism, preferentially promoting subcutaneous fat deposition while attenuating visceral fat accumulation among those who are premenopausal.^[24]^ This hormonal influence serves as a protective mechanism against central obesity development. Moreover, the cardiovascular protective effects of estrogen, such as improved endothelial function and increased HDL cholesterol levels, collectively contribute to the relatively lower risk of hypertension observed in premenopausal females with low-WHtR compared to their male counterparts.^[25–27]^

Progression to central adiposity induces a series of pathological changes, including aggravated insulin resistance, enhanced secretion of inflammatory adipokines such as resistin and leptin, and increased activation of the renin-angiotensin-aldosterone system.^[28]^ This metabolic imbalance leads to impaired endothelial function and a subsequent rise in blood pressure.^[29]^ The transition from healthy to central adiposity may precipitate a more pronounced elevation in hypertension risk among premenopausal females, potentially due to the disruption of established estrogen-mediated protective mechanisms.

In females over 60, the considerable decline in the protective effects of estrogen following menopause significantly increases the risk of visceral fat accumulation and cardiovascular damage, resembling patterns observed in males.^[30]^ These changes may explain the similar patterns of hypertension prevalence across central adiposity groups in postmenopausal females and males.

Importantly, in South Korea, there has been a shift towards a more westernized lifestyle, including increased consumption of refined carbohydrates and high-calorie diets, and a trend towards physical inactivity.^[3,31]^ These changes may have been a major factor in accelerating the accumulation of abdominal fat and simultaneously increasing the risk of hypertension.^[32]^ This may partly explain the more pronounced increase in hypertension among the high-WHtR group.

### 4.4. Clinical and policy implications

Healthcare providers in both hospital and primary care settings should prioritize proactive diagnosis and management of central obesity. Early detection is critical for preventing or mitigating associated risks, particularly hypertension.^[33]^ Because the risk and progression of central obesity differ by sex and age, clinicians should adopt tailored prevention and treatment strategies.^[23]^ This includes incorporating routine assessments of WHtR as a superior predictor of hypertension risk compared to BMI, enabling more precise identification of at-risk individuals.

Additionally, healthcare providers should address modifiable lifestyle factors, such as promoting physical activity, advocating for balanced dietary habits, and counseling patients on stress management techniques.^[1]^ These interventions are especially important for high-risk groups, such as middle-aged women transitioning into menopause, who may experience rapid changes in fat distribution and associated hypertension risk.

Public health authorities should broaden screening and management programs for central obesity, particularly targeting middle-aged and older adults who exhibit elevated rates of hypertension.^[9]^ Such measures could contibute to national initiatives such as South Korea’s National Health Plan 2030, which seeks to sustain the current adult prevalence of abdominal obesity through 2030.^[34]^

### 4.5. Strength and limitations

The findings of this study should be interpreted with consideration of its limitations. Given the cross-sectional study of the KNHANES data, the conclusion of our study is limited to draw causal inferences. This limitation should be acknowledged, and future longitudinal cohort studies or interventional trials are required to clarify causal relationships. However, by using a dataset representative of the Korean population with a large sample size of 98,396 participants, the findings are more generalizable and provide robust insights into trends and associations. Second, individuals aged 0 to 29 years were excluded from the analysis, resulting in a lack of data for younger individuals. However, this focus aligns with the standard age range for adult hypertension research, facilitating precise analysis and appropriate contextual interpretation.^[1,11]^ Third, the use of self-reported data may have introduced potential recall bias, which could affect the accuracy of the findings. Lastly, the study exclusively focused on the Korean population, reflecting the sociocultural context of Korea. Consequently, the results may not be directly applicable to global trends or other populations.

Despite these limitations, this study has notable strengths. It utilizes large-scale nationally representative data to provide insights into hypertension prevalence trends stratified by central obesity levels and the associated risk factors. Furthermore, it emphasizes the differences in the association between central obesity and hypertension across sex and age groups, highlighting the importance of identifying and managing risk factors for hypertension.

## 5. Conclusion

This study investigated that the prevalence of hypertension increased from 2005 to 2019, with a more pronounced increase observed in the high-WHtR group. Among individuals in the low-WHtR group, males showed a significantly higher prevalence of hypertension compared to females. However, in the high-WHtR group, the prevalence difference between sexes was relatively smaller. Additionally, subgroup analyses revealed that females aged 30 to 59 years with high-WHtR had a higher risk of hypertension, whereas males aged 60 years or older demonstrated a greater risk. Our findings highlight the importance of using WHtR for the effective management of central obesity, with particular emphasis on age- and sex-specific approaches, and the expansion of targeted screening programs.

## Author contributions

**Conceptualization:** Jaehyun Kong, Hyeseung Lee, Hyunjee Kim, Jiyoung Hwang, Dong Keon Yon.

**Data curation:** Jaehyun Kong, Hyeseung Lee, Hyunjee Kim, Jiyoung Hwang, Dong Keon Yon.

**Formal analysis:** Jaehyun Kong, Hyeseung Lee, Hyunjee Kim, Jiyoung Hwang, Dong Keon Yon.

**Funding acquisition:** Jaehyun Kong, Hyeseung Lee, Hyunjee Kim, Jiyoung Hwang, Dong Keon Yon.

**Investigation:** Jaehyun Kong, Hyeseung Lee, Hyunjee Kim, Jiyoung Hwang, Dong Keon Yon.

**Methodology:** Jaehyun Kong, Hyeseung Lee, Hyunjee Kim, Jiyoung Hwang, Dong Keon Yon.

**Project administration:** Jaehyun Kong, Hyeseung Lee, Hyunjee Kim, Jiyoung Hwang, Dong Keon Yon.

**Resources:** Jaehyun Kong, Hyeseung Lee, Hyunjee Kim, Jiyoung Hwang, Dong Keon Yon.

**Software:** Jaehyun Kong, Hyeseung Lee, Hyunjee Kim, Jiyoung Hwang, Dong Keon Yon.

**Supervision:** Dong Keon Yon.

**Validation:** Jaehyun Kong, Hyeseung Lee, Hyunjee Kim, Jiyoung Hwang, Dong Keon Yon.

**Visualization:** Jaehyun Kong, Hyeseung Lee, Hyunjee Kim, Jiyoung Hwang, Dong Keon Yon.

**Writing – original draft:** Jaehyun Kong, Hyeseung Lee, Hyunjee Kim, Jiyoung Hwang, Dong Keon Yon.

**Writing – review & editing:** Jaehyun Kong, Hyeseung Lee, Hyunjee Kim, Yesol Yim, Sooji Lee, Seohyun Hong, Lee Smith, Damiano Pizzol, Jaewon Kim, Ho Geol Woo, Jiyoung Hwang, Dong Keon Yon.

## Supplementary Material


