# Supplementary material for: National trends in hypertension stratified by central adiposity using waist-to-height ratio in South Korea, 2005 to 2022
Source: Medicine (Baltimore). 2025 Aug 15;104(33):e43773. doi: 10.1097/MD.0000000000043773 (PMC12366897; doi:10.1097/MD.0000000000043773)
Supplement: Supplementary file 1 [file medi-104-e43773-s001.docx]

| **Supplement Material** |
| --- |

Original Article

National trends in hypertension stratified by central adiposity using waist-to-height ratio in South Korea, 2005–2022

Running title: Hypertension trend by central adiposity

Jaehyun Kong MD^a,b†^, Hyeseung Lee MD^a,b†^, Hyunjee Kim MS^a,c3†^, Yesol Yim MS^a,c^, Sooji Lee MD^a,b^, Seohyun Hong MD^a,b^, Lee Smith PhD^d,e^, Damiano Pizzol MD, PhD^f,g^, Jaewon Kim MD, PhD^a^, Ho Geol Woo MD, PhD^h^, Jiyoung Hwang PhD^a,b^, Dong Keon Yon MD, PhD^a,b,c,i*^

^†^These authors were contributed equally as first authors.

*Corresponding author:

**Dong Keon Yon**, MD, PhD, FACAAI, FAAAAI, ATSF (lead contact)

Department of Pediatrics, Kyung Hee University College of Medicine, 23 Kyungheedae–ro, Dongdaemun–gu, Seoul 02447, South Korea

Tel: +82–2–6935–2476

Fax: +82–504–478–0201

Email: [yonkkang@gmail.com](mailto:yonkkang@gmail.com)


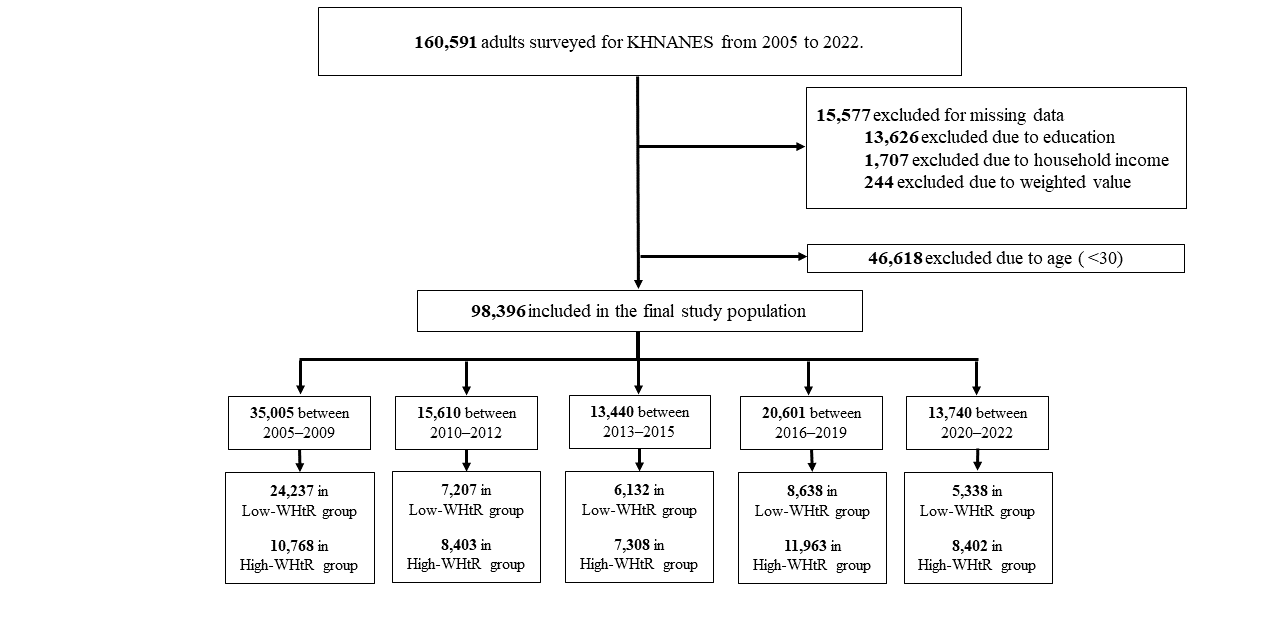
**Figure S1.** Study population in KNHANES, 2005–2022.

*Abbreviations*: KNHANES, Korea National Health and Nutrition Examination Survey.
